# Red flags to suspect inborn errors of immunity in patients with autoimmune diseases

**DOI:** 10.7705/biomedica.7561

**Published:** 2024-12-23

**Authors:** Natalia Vélez, Juliette De Ávila, Jaime Cortés, Nelson Barrero, Leosirlay Rojas, Juan Manuel Bello, Consuelo Romero-Sánchez

**Affiliations:** 1 Grupo de Inmunología Celular y Molecular - InmuBo, Universidad El Bosque, Bogotá, D. C., Colombia Universidad El Bosque Grupo de Inmunología Celular y Molecular - InmuBo Universidad El Bosque Bogotá, D. C. Colombia; 2 Inmunología Clínica y Alergia Pediátrica, Fundación Hospital Pediátrico La Misericordia, Bogotá, D. C., Colombia Fundación Hospital Pediátrico La Misericordia Inmunología Clínica y Alergia Pediátrica Fundación Hospital Pediátrico La Misericordia Bogotá, D. C. Colombia; 3 Medicina Interna, Escuela de Medicina, Universidad Militar Nueva Granada, Bogotá, D. C., Colombia Universidad Militar Nueva Granada Medicina Interna, Escuela de Medicina Universidad Militar Nueva Granada Bogotá, D. C. Colombia; 4 Reumatologia, Escuela de Medicina, Universidad Militar Nueva Granada, Bogotá, D. C., Colombia Universidad Militar Nueva Granada Reumatologia, Escuela de Medicina Universidad Militar Nueva Granada Bogotá, D. C. Colombia; 5 Reumatologia Pediátrica, Facultad de Medicina, Universidad El Bosque, Bogotá, D. C., Colombia Universidad El Bosque Reumatologia Pediátrica, Facultad de Medicina Universidad El Bosque Bogotá, D. C. Colombia; 6 Grupo de Inmunología, Escuela de Medicina, Universidad Militar Nueva Granada, Bogotá, D. C., Colombia Universidad Militar Nueva Granada Grupo de Inmunología, Escuela de Medicina Universidad Militar Nueva Granada Bogotá, D. C. Colombia; 7 Departamento de Reumatologia e Inmunología, Hospital Militar Central, Bogotá, D. C., Colombia Hospital Militar Central Departamento de Reumatologia e Inmunología Hospital Militar Central Bogotá, D. C. Colombia

**Keywords:** Autoimmunity, autoimmune diseases, early-onset immune dysregulation, cytopenia, anemia, hemolytic, autoimmune, thrombocytopenia, lupus erythematosus, systemic, inflammatory bowel disease., autoinmunidad, enfermedades autoinmunitarias, citopenia, anemia hemolítica autoinmunitaria, trombocitopenia, lupus eritematoso sistémico, enfermedades inflamatorias intestinales.

## Abstract

Inborn errors of immunity are monogenic disorders that predispose patients to immune dysregulation, autoimmunity, and infection. Some autoimmune diseases, such as autoimmune cytopenias, systemic lupus erythematosus, and inflammatory bowel diseases, are increasingly recognized as phenotypes of inborn errors of immunity.

The objective of this article was to identify red flags or clinical/laboratory markers to suspect inborn errors of immunity in patients with autoimmune cytopenias, systemic lupus erythematosus, and inflammatory bowel diseases through a systematic literature review. The study followed the systematic reviews and meta-analysis guidelines (PRISMA). After selection, we included 36 articles, and their methodological quality was verified using the Joanna Briggs Institute tools for individual risk of bias analysis.

The principal red flags in autoimmune cytopenias are chronic, recurrent, and refractory cytopenias, recurrent infection, severe infectious complications associated with immunosuppressive treatment, and chronic lymphoproliferation. In systemic lupus erythematosus, red flags include age of onset before five years, severe organ involvement, chilblain lesions, and chronic lymphoproliferation. For inflammatory bowel diseases, red flags are an age of onset before two years, resistance to conventional therapies, atypical endoscopic or histologic findings, and consanguineous parents. Autoimmune diseases may be the primary manifestation of inborn errors of immunity in pediatric and adult patients. An early diagnosis of a monogenic disorder allows for the tailoring of effective treatment plans, providing prognostic information to families, and offering genetic counseling.

## Resumen

Los errores innatos de la inmunidad son trastornos monogénicos que predisponen a los pacientes a la desregulación inmunitaria, la autoinmunidad y las infecciones. Algunas enfermedades autoinmunitarias, como las citopenias autoinmunitarias, el lupus eritematoso sistémico y las enfermedades inflamatorias intestinales son cada vez más reconocidas como errores innatos de la inmunidad.

El objetivo de este artículo fue investigar las señales de alerta clínicas o de laboratorio que pueden hacer sospechar errores innatos de la inmunidad en pacientes con citopenias autoinmunitarias, lupus eritematoso sistémico y enfermedades inflamatorias intestinales, mediante una revisión sistemática de la literatura.

El estudio se realizó bajo los parámetros para revisiones sistemáticas y metaanálisis (PRISMA). Después de la selección, se incluyeron 36 artículos cuya calidad metodológica se verificó por medio de las herramientas para el análisis individual del riesgo de sesgo del *Joanna Briggs Institute*.

Las principales señales de alerta en las citopenias autoinmunitarias son presentación crónica, recurrente y resistente al tratamiento, infecciones recurrentes, complicaciones infecciosas graves asociadas con el tratamiento inmunosupresor y linfoproliferación crónica. En el lupus, las señales de alerta incluyen una edad de inicio antes de los 5 años, compromiso grave de órgano blanco, perniosis y linfoproliferación crónica. Finalmente, las señales de alerta en la enfermedad inflamatoria intestinal son la edad de inicio antes de los 2 años, la resistencia a los tratamientos convencionales, los hallazgos atípicos en la endoscopia o la histología, y la consanguinidad en los padres.

Las enfermedades autoinmunitarias pueden ser la primera manifestación de los errores innatos de la inmunidad. El diagnóstico temprano de un defecto monogénico permite iniciar oportunamente el tratamiento farmacológico dirigido, ofrecer información sobre el pronóstico y brindar asesoría genética.

Inborn errors of immunity represent a heterogeneous group of diseases resulting from inherited defects in immune cell development, maturation, and function. Inborn errors of immunity often have a substantial genetic basis, leading to various immune disorders associated with infections, autoimmune or allergic diseases, and an increased risk of malignancies. As these are congenital conditions, usually with well-defined genetic defects and Mendelian inheritance, children are the most predominant patients. To date, more than 430 monogenic traits falling under inborn errors of immunity have been reported ^1^.

Inborn errors of immunity include many disorders affecting different components of innate and adaptive immune responses. Advances in genetics and a greater understanding of the pathophysiological processes involving B and T-cell development and signaling, immune tolerance, complement pathways, and inflammation demonstrate how inborn errors of immunity and autoimmunity are interconnected, sharing common mechanisms ^2^.

Tolerance processes are fundamental in the development of autoimmune diseases. Random recombination events during thymic T-cell development yield a broad T-cell repertoire. Central T-cell tolerance is achieved through negative selection, whereby thymocytes recognizing self-antigens -displayed on major histocompatibility complex (MHC) molecules of medullary epithelial cells or thymic dendritic cells with higher affinity- undergo clonal deletion. Autoreactivity may, to some extent, be physiological, participating in the necessary process called positive selection of lymphocytes and homeostasis of the immune system. An alternative fate for autoreactive thymocytes is their differentiation into natural or constitutive CD4+/CD25+/FOXP3+ regulatory T cells, which can suppress the induction and activation of effector T cells, preventing or regulating immune responses ^3^.

In contrast, aberrant responses to self-antigens underlie more than 80 inflammatory conditions, defined as autoimmune diseases, a heterogeneous group of disorders of the immune system secondary to a loss of tolerance to self-antigens that may be organ-specific or systemic, such as rheumatoid arthritis, Sjogren’s syndrome, and systemic lupus erythematosus. They appear to have a polygenic nature and are often the consequence of a pathogenic interplay between environmental and genetic factors ^4^. Extensive cohort studies detail the clinical spectrum and treatment outcomes of inborn errors of immunity with autoimmunity involving specific immune genes (*e.g., CTLA4, LRBA, PI3Kδ, NFkβ1, RAG*). Early recognition of the monogenic origin of an autoimmune disease allows prompt initiation of targeted therapies to restore immune balance through medications (such as biologic products) or hematopoietic stem cell transplant ^5^.

Therefore, the following question arises: When must we suspect an inborn error of immunity in a patient with an autoimmune disease? Most of the available information comes from patients with inborn errors of immunity who frequently have autoimmune manifestations. In our clinical experience, the most frequent manifestations are inflammatory bowel disease, autoimmune hemolytic anemia, autoimmune thrombocytopenia, and lupus-like disease.

For this reason, this systematic review aimed to identify the red flags or clinical and laboratory markers to suspect inborn errors of immunity in patients with specific autoimmune diseases.

## Materials and methods

The systematic review was carried out according to the guidelines of systematic reviews and meta-analyses (PRISMA). This structured guide includes recommendations based on 27 questions to facilitate the preparation of systematic reviews and meta-analyses, proposing methods to synthesize and present findings reproducibly and with quality ^6^.

### Research question based on the PEPOH structure

The research question was formulated according to the PEPOH structure:

- *Population (P)*: Individuals with chilblain lupus, systemic lupus erythematosus, inflammatory bowel disease, polyautoimmunity, autoimmune hemolytic anemia, thrombocytopenic purpura, or Evans’ syndrome.
- *Exposure (E)*: Altered genes or metabolites associated with inborn errors of immunity.
- *Target population (P)*: The scientific community and health professionals.
- *Outcomes (O)*: Evidence of misdiagnosis or misclassification of diseases.
- *Health care setting (H)*: Community; primary, secondary, and tertiary level health care settings.

### Eligibility criteria

#### Inclusion criteria

- Studies in English or Spanish available in full text.
- Study population: Subjects with chilblain lupus, systemic lupus erythematosus, inflammatory bowel diseases, polyautoimmunity, autoimmune hemolytic anemia, thrombocytopenic purpura, or Evans syndrome.
- Types of studies: Observational (descriptive such as cross-sectional, case report, and case series studies) and analytical (case-control, prospective, and retrospective cohort studies).

#### Exclusion criteria

- Studies on other rheumatic diseases, such as rheumatoid arthritis or psoriasis.
- Studies inborn errors of immunity diagnosed before the autoimmune disease.

### Outcome measures

- Misclassification bias, differential misclassification, non-differential misclassification, misdiagnosis, or late diagnosis of inborn errors of immunity.

### Sources of information

- Databases: Embase and PubMed.

### Search strategy

- Generic search in databases without time restriction using controlled terms (MeSH, Emtree) and free-term language. The connectors used to facilitate and improve the search were: “AND” and “OR”. A search strategy was established by combining key terms with the connectors for each database. Additionally, a search for related articles was conducted using the forward and reverse snowball strategy with the papers selected in the initial stage.

### Screening and study selection

#### Selection of articles

The search and selection of the articles were carried out per duplicate, with two researchers independently conducting it two stages. The first stage involved an initial screening according to title and abstract, discarding those not meeting the eligibility criteria according to language and population. Disagreements between the evaluators were resolved by consensus without requiring a third evaluator. In the second stage, the full text of the studies selected in the first stage was evaluated independently by the researchers, reaching agreements without requiring a third evaluator. Duplicate articles were discarded before screening.

### Assessment of methodological quality

A single researcher assessed the quality of the selected studies using the critical appraisal tools provided for systematic reviews by the Joanna Briggs Institute. These tools allowed us to evaluate the risk of bias in the included articles according to a checklist for each type of study (case report, case series, case-control, cohort, and cross-sectional studies) ^7^^,^^8^.

### Data extraction and analysis

The articles selected for further analysis were classified into five groups according to the type of study and analyzed in a descriptive table. Two investigators independently assessed the studies retrieved by the search strategy and excluded them based on titles, abstracts, or both. The same authors independently reviewed the studies selected for full-text analysis. One author extracted the data into a collection matrix on a spreadsheet. In cases of disagreement between the investigators, the data were reviewed until reaching a consensus. The intervention of other evaluators was not necessary.

The narrative synthesis was presented through tables of findings according to the data collected in the Excel sheet, classified by the type of studied population. The collected data included the following variables: type of study, sample size and population, methods, results, conclusions, contributions, practical implications, and limitations.

## Results

### Selection of articles

**Figure 1.**
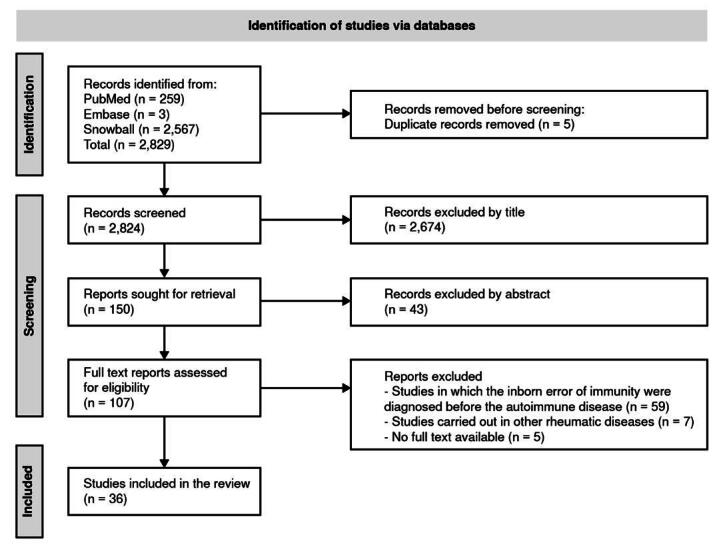
The PRISMA flow diagram for the systematic review detailing the database searches, the number of screened abstracts, and the retrieved full-texts

We obtained 262 articles from the databases (PubMed: 259, Embase: 3), while with the snowball search strategy (forward and reverse method), we found 2,567 records. Finally, 2,829 articles were identified before excluding duplicates (n = 5). During the screening process, we excluded 2,717 articles by title and abstract. Based on the full-text evaluation, we discarded 71 articles. Ultimately, we selected 36 manuscripts: 17 from the initial search and 19 from the snowball strategy (figure 1).

### Data extraction

The selected articles were classified into three groups according to diagnosis: 1) autoimmune cytopenias or Evans’ syndrome, 2) systemic lupus erythematosus, and 3) inflammatory bowel diseases. All grouped papers were analyzed in a descriptive table (table 1).

**Table 1.** Summary of studies in autoimmune diseases with clinical features associated with inborn errors of immunity published between 2014 and 2024

Author (year)	Type of study	Population	Methods	Results	Conclusions	Contributions	Practical implications	Limitations
Cytopenias and inborn errors of immunity in adult and pediatric populations								
Fioredda *et al.* (2020)	Retrospective cross-sectional study	The study included 214 patients with autoimmune neutropenia from the registry.	Defined autoimmune neutropenia with absolute neutrophil count threshold, lasting more than three months, and positive antibody testing. Used GIFT (granulocyte immunofluorescence test) for antibody testing, centralized in one laboratory.	Long onset-neutropenia shows more autoimmune signature than long-lasting neutropenia. Long onset-neutropenia may indicate immune deficiency or dysregulation.	Late onset-neutropenia may indicate future immune deficiency or dysregulation. Late onset-neutropenia requires long-term monitoring and extensive immunologic workup. Early diagnosis of immunologic disease can prevent complications and enable targeted therapies.	Designing research on autoimmune neutropenia Performing laboratory analysis and data collection Reviewing and analyzing data for autoimmune neutropenia research	Early diagnosis aids in preventing complications and enables targeted therapies. Extensive immune investigations are crucial for cases with late onset-neutropenia.	Selection bias and incomplete data due to retrospective registry analysis. Short follow-up may miss patients transitioning to immunodeficiency.
Miaño *et al.* (2022)	Retrospective cross-sectional study	The sample size in the study was 40 patients with Evans’ syndrome: 23 men and 17 women included in the analysis.	Genetic studies with next generation sequencing analysis of 315 genes related to disorders	Genetic variants found in 45% of patients with autoimmune lymphoproliferative syndrome. Patients with pathogenic variants required more second-line therapies. Stem cell transplantation only for patients with genetic variants	Evans’ syndrome and multilineage autoimmune cytopenias are epiphenomena of underlying inborn errors of immunity in patients. Genetic screening is crucial for diagnosis and tailored treatments. Immune dysregulation in patients with multilineage cytopenia required specific follow-up. Specific monogenic defects enable targeted therapies for patients.	Evaluating genetic background and clinical features of pediatric patients with Evans’ syndrome Addressing genetic and immunological differences between classical Evans’ syndrome and multilineage cytopenia	Genetic screening is essential for Evans’ syndrome and multilineage autoimmune cytopenia. patients for specific treatments Immune dysregulation in patients requires alert and specific follow-up. Multilineage cytopenia can be an epiphenomenon of underlying disorders.	Limited detection rate due to varied genetic testing approaches Potential need for deeper genetic investigation beyond next generation sequencing analysis
Kumar *et al.* (2021)	Case-control study	The study involved 24 patients with pediatric Evans’ syndrome: 22 with chronic immune thrombocytopenia, and 24 healthy controls.	High-dimensional immunophenotyping, gene expression analysis, cytokine profiling, TCR-(3 repertoire studies	Expanded circulating follicular helper T (cTfh), decreased naive CD4, increased T-cell activation in pediatric Evans’ syndrome Increased cTfh percentage, activation markers, and T-cell activation in pediatric Evans’ syndrome Decreased class-switched memory B cells in most patients with pediatric Evans’ syndrome Broad immune abnormalities in pediatric Evans’ syndrome differ from chronic immune thrombocytopenia.	Expansion of cTfh and T-cell activation are key pediatric Evans’ syndrome features. Immune abnormalities in pediatric Evans’ syndrome differ significantly from chronic immune thrombocytopenia.	Identified genetic defects in immune regulatory pathway in patients with pediatric Evans’ syndrome. Described immune anomalies in patients with pediatric Evans’ syndrome.	Characterizes immune abnormalities in pediatric Evans’ syndrome. Identified cTfh expansion, T-cell activation, and B-cell defects.	Lack of defining immune markers for pediatric Evans’ syndrome Poor understanding of immune abnormalities in pediatric Evans’ syndrome
Karalexi *et al.* (2018)	Case-report study		Sequencing analysis confirmed Wiskott- Aldrich syndrome Flow cytometric analysis of intracellular *WASp* expression was performed.	Misdiagnosed Wiskott- Aldrich syndrome as autoimmune thrombocytopenia in a 3-year-old boy. Confirmed Wiskott-Aldrich syndrome diagnosis through sequencing analysis Patient planned for stem cell transplantation due to refractory treatment	Misdiagnosis of Wiskott- Aldrich syndrome as autoimmune thrombocytopenia raised awareness for males. Hematopoietic stem cell transplantation is the optimal treatment for Wiskott-Aldrich syndrome with curable outcomes. Wiskott-Aldrich syndrome should be considered in males with chronic autoimmune thrombocytopenia-like features. *WASp* gene mutations can induce different clinical outcomes. Early diagnosis and monitoring are crucial for improving patient’s outcomes.	Raised awareness on misdiagnosis of Wiskott-Aldrich syndrome in males. Highlighted the importance of considering Wiskott-Aldrich syndrome in immune thrombocytopenic purpura cases.	Early Wiskott-Aldrich syndrome diagnosis is crucial for appropriate treatment and management. Consider Wiskott- Aldrich syndrome in males with infant-onset thrombocytopenia-like features. *WASp* expression analysis and sequencing is essential for Wiskott-Aldrich syndrome diagnosis.	Wiskott-Aldrich syndrome diagnosis may be challenging due to multifaceted nature. Delayed diagnosis in children with milder phenotypes like X-linked thrombocytopenia
Kedar *et al.* (2022)	Case-report study		Targeted next generation sequencing (t-NGS) for LRBA gene mutations Bioinformatics analysis and protein modeling for novel mutations	Novel LRBA gene mutation causing severe hemolysis in an Indian family Timely targeted exorne sequencing for unexplained hemolytic anemia diagnosis	Identified LRBA variant explains severe hemolytic anemia in the family. Timely exorne sequencing aids in diagnosing hemolytic anemia with varied phenotypes.	Unusual LRBA deficiency case with severe anemia and jaundice An identified novel homozygous mutation causes severe hemolysis in an Indian family.	Timely exorne sequencing aids in diagnosing unexplained haemolytic anaemia. Diagnosis of LRBA deficiency by targeted next generation sequencing technology	No specific limitations were mentioned in the provided context.
Zama *et al.* (2021)	Retrospective cross-sectional study	The data were collected from 47 pediatric patients with at least one hematological disorder, forming the analyzed cohort.	Division of patients into those with inborn errors of immunity and those without them based on their diagnoses Extended immunophenotyping to identify specific characteristics in the patients with inborn errors of immunity Molecular analysis using a targeted gene panel specific for inborn errors of immunity	Inborn errors of immunity prevalence in cytopenias: autoimmune thrombocytopenia (42%), autoimmune hemolytic anemia (64%), autoimmune neutropenia (36%), Evans syndrome (62%) Inborn errors of immunity characteristics: T or B lymphopenia, decreased memory B-cells, decreased plasmablasts Common variable immunodeficiency patients: 2/9 had molecular diagnosis for inborn errors of immunity.	Strong association between immune cytopenias and inborn errors of immunity in pediatric patients Extended immunophenotyping useful for detecting inborn errors of immunity signs in immune cytopenic children Identified specific characteristics associated with inborn errors of immunity in pediatric patients.	Study design, data collection, statistical analysis, and molecular analyses were key.	Prompt identification of children with immune cytopenias needing genetic evaluation. Extended lymphocyte immunophenotyping is useful for detecting inborn errors of immunity.	Sample size and retrospective nature are primary limitations. High diagnosis rate may be due to tertiary care center enrollment.
Azizi *et al.* (2021)	Retrospective cross-sectional study	The sample size included 471 patients with primary antibody deficiencies.	Flow cytometry analysis for Tregs count and assessment Antibody response measurement using enzyme-linked immunosorbent assay -Autoimmunity diagnosis based on clinical and paraclinical findings	Autoimmunity assessment in patients with primary antibody deficiencies, frequent in those with common variable immunodeficiency and gastrointestinal manifestations - Clinical insights for managing autoimmunity in patients with primary antibody deficiencies, emphasizing in timely immunoglobulin replacement therapy	Study enhances understanding of autoimmunities in primary antibody deficiencies. Emphasizes the importance of early diagnosis and timely immunoglobulin replacement therapy.	- Described clinical features of patients with primary antibody deficiencies and autoimmune manifestations. - Compares features among common variable i m mu nodef ici ency, agammaglobulinemia, symptomatic sIgAD, and CSD.	Enhances understanding of autoimmunities in primary antibody deficiencies. Aids in managing and treating patients with primary antibody deficiencies.	Limited focus on treatment outcomes for autoimmune disorders Lack of detailed discussion on the impact of autoimmunity progression
Schiavo *et al.* (2022)	Prospective study	The study included 22 participants, with 7 patients with autoimmune cytopenias (alone) and 15 patients with autoimmune cytopenias and immune errors of immunity.	Data collection, immunophenotyping analysis, and genetic analysis	Dysregulated immunophenotype identified key inborn errors of immunity-associated alterations for early diagnosis. T cell subpopulations analysis showed differences between the two groups of patients (autoimmune cytopenias with or without inborn errors of immunity).	Dysregulated immunophenotype contributes to early inborn errors of immunity diagnosis for appropriate clinical management. Autoimmune cytopenias can be red flags for inborn errors of immunity in pediatric patients. T cell subpopulation analysis is crucial for patients with autoimmune cytopenias with or without inborn errors of immunity	Investigated immunological and genetic background of pediatric patients with autoimmune cytopenias. Identified key inborn errors of immunity- associated alterations causative of specific diseases. Used immune phenotyping as a screening tool for early inborn errors of immunity diagnosis.	Early inborn errors of immunity diagnosis through dysregulated immunophenotype for better clinical management Genetic analysis for inborn errors of immunity-associated alterations in patients with refractory autoimmune cytopenias	Limited data sharing due to ethical restrictions on raw datasets Inconclusive genetic analysis for some patients with autoimmune cytopenias and inborn errors of immunity requiring further investigations
Samitinjay *et al.* (2022)	Case-report study		Clinical history review Physical examination Diagnostic tests for autoimmune hemolytic anemia	Patient diagnosed with autoimmune hemolytic anemia due to autoimmune manifestation of a primary immunodeficiency Treatment included blood transfusion, dexamethasone injections, and supportive therapy. Patient’s symptoms resolved post-transfusion, with improved hemoglobin levels.	Diagnostic challenges in autoimmune hemolytic anemia due to the immunodeficiency discussed. Proposal for feasible solutions to tackle diagnostic and therapeutic challenges.	Analysis of data discontinuity and importance of well-kept medical records Diagnostic and therapeutic challenges faced by the patient and healthcare system	Proposes solutions for diagnostic and therapeutic challenges in immunodeficiency disorders. Highlights the need for centralized and accessible electronic medical records. Questions the efficacy of immunoglobulin replacement therapy in common variable immunodeficiency patients.	Electronic health records may lead to data breaches and doctor burnout. Electronic health records may not improve healthcare delivery due to software limitations.
Dutmer *et al.* (2015)	Case-report study		Next-generation sequencing of genomic DNA for RAG2 mutations Immunophenotyping of peripheral blood lymphocytes and immune function studies Analysis of recombination activity of wild-type and mutant RAG2 constructs Lymphocyte immunophenotyping, proliferation assays, and toll-like receptor ligand- induced TNF production	Virus genotyping revealed vaccine-strain VZV, mumps, and rubella. Next generation sequencing identified heterozygous variants R73H and P180H for RAG2. Residual RAG2 protein activity was demonstrated despite hypomorphic mutations.	Late-onset RAG2 mutations led to fatal vaccine-strain VZV infection. Live virus vaccination and immunosuppressive therapies can have negative consequences Corticosteroid therapy in RAG2 deficiency resulted in profound lymphopenia.	Identifying novel defects in known genes expanded clinical disease spectrum Clinical suspicion for RAG mutations is crucial to avoid complications.	Late-onset RAG2 deficiency can lead to severe vaccine-strain infections. Corticosteroid therapy can exacerbate immune system compromise in RAG2 deficiency. Hypomorphic RAG2 mutations may delay immune deficiency identification.	Lack of newborn screening for severe combined immunodeficiency in RAG2 deficient patient Delayed identification of immune deficiency due to hypomorphic RAG2 mutations
Arays *et al.* (2016)	Case-report study		Genetic studies not required for common variable immunodeficiency diagnosis Flow cytometry for B cell immunophenotyping to identify patient subgroups	Diagnosis of common variable immunodeficiency in a postpartum female with recurrent infections Importance of early diagnosis and immunoglobulin therapy for common variable immunodeficiency patients	Early common variable immunodeficiency diagnosis is crucial to prevent morbidity. Immunoglobulin levels are vital before splenectomy and rituximab treatment. Genetic studies are not mandatory for common variable immunodeficiency diagnosis.	Varied common variable immunodeficiency presentation reminder Diagnostic criteria review Rituximab and splenectomy use in primary immunodeficiency	Early common variable immunodeficiency diagnosis is crucial to prevent serious infections. Immunoglobulin replacement therapy is essential for common variable immunodeficiency management.	Lack of genetic studies for common variable immunodeficiency diagnosis High cost of immunoglobulin replacement therapy
Dekeyser *et al.* (2022)	Case-report study		Flow cytometry on peripheral blood to elevate T lymphocytes Inborn error of immunity gene panel to identify FAS gene variants	Patient showed longitudinal extensive transverse myelitis with multiple contrast-enhancing lesions on magnetic resonance imaging. Elevated double negative T lymphocytes confirmed autoimmune lymphoproliferative syndrome diagnosis. Autoimmune hemolytic anemia developed after rituximab treatment, resolved with methylprednisolone.	Autoimmune lymphoproliferative syndrome should be suspected in non-malignant lymphoproliferation with autoimmunity. Gene panels aid in screening for a wide range of inborn errors of immunity causes. Neurological symptoms in recurrent infections warrant evaluation for inborn errors of immunity.	Described a unique case of longitudinal extensive transverse myelitis in autoimmune lymphoproliferative syndrome. Highlighted diagnostic clues for autoimmune lymphoproliferative syndrome and the need for further testing. Emphasized the importance of considering a broad differential diagnosis for inborn errors of immunity. Discussed the role of gene panels in screening for inborn errors of immunity causes.	Autoimmune lymphoproliferative syndrome should be suspected in patients with lymphoproliferation and neurological manifestations. Gene panels aid in screening for a wide range of inborn errors of immunity. Neurological symptoms in recurrent infections warrant inborn errors of immunity evaluation.	Limited reported cases of autoimmune lymphoproliferative syndrome-related disorders with longitudinal extensive transverse myelitis Autoimmune lymphoproliferative syndrome-like disorders have distinct genetic features but limited longitudinal extensive transverse myelitis cases.
Dell’Orso *et al.* (2021)	Case-report study		Next-generation sequencing panel for genetic diagnosis Functional assay on peripheral monocytes to test ADA2 activity	Patient had two novel ADA2/CECR1 gene mutations. Genetic diagnosis crucial for precision medicine and targeted treatments Patient’s clinical history fulfilled 2009 NIH autoimmune lymphoproliferative syndrome criteria.	Early genetic evaluation is crucial for immune dysregulation and marrow failure diagnosis. Precision medicine approach with targeted treatments is recommended for ADA2 deficiency. Genetic diagnosis aids to define tailored treatment or hematopoietic stem cell transplantation.	Clinical onset of ADA2 deficiency and autoimmune lymphoproliferative syndrome features reported. Genetic diagnosis importance for precision medicine and targeted treatments Functional assay of ADA2 activity conducted.	Early genetic diagnosis guides precision medicine and targeted treatments. Improved diagnostic accuracy leads to early targeted therapy options.	Limited understanding of ADA2 deficiency pathogenesis and marrow failure. Unclear residual enzymatic activity impact on vasculitis and marrow failure.
Martínez- Valdez *et al.* (2021)	Case-series study	Three pediatric patients with corticosteroid- refractory Evans’ syndrome who were ultimately diagnosed with common variable immunodeficiency.	Immune evaluation included immunoglobulin levels, subclasses, and antibody testing. Genetic studies were conducted when necessary.	Evans’ syndrome patients diagnosed with common variable immunodeficiency showed improvement with specific therapy. Detection of primary immunodeficiencies in Evans’ syndrome cases is crucial for effective treatment. Gene-related forms of common variable immunodeficiency like CTLA4 and LRBA deficiencies identified Patients with Evans’ syndrome ultimately diagnosed with common variable immunodeficiency without significant infections	Detection of primary immunodeficiencies in Evans’ syndrome cases is crucial for optimal treatment. Common variable immunodeficiency-specific therapy is essential for managing cytopenias in these cases. Gene-related forms of common variable immunodeficiency like CTLA4 and LRBA deficiencies identified Importance of detecting common variable immunodeficiency patients from those with autoimmune cytopenias	Detection of primary immunodeficiency in Evans’ syndrome cases Importance of common variable immunodeficiency treatment for controlling autoimmune cytopenias Identification of gene-related forms of common variable immunodeficiency in pediatric patients	Early primary immunodeficiency detection is crucial for Evans’ syndrome patients’ optimal management. Common variable immunodeficiency- specific therapy is essential for controlling autoimmune cytopenias in Evans’ syndrome patients. Genetic studies may reveal underlying primary immunodeficiencies in Evans’ syndrome patients.	Lack of genetic mutations in known primary immunodeficiencies genes in some patients Reliance on corticosteroids for disease control in some cases Occurrence of new relapses despite various treatment strategies
Besnard *et al.* (2018)	Retrospective cross- sectional study	The study evaluated 48 patients with Evans’ syndrome over an 18-year period. After excluding 30 patients with autoimmune lymphoproliferative syndrome, the study included 18 patients for genetic testing.	Clinical records review Genetic testing for primary immunodeficiencies gene defects Sanger and next generation sequencing for mutation identification	Gene defects identified in seven patients: CTLA4, LRBA, KRAS, STAT3 No significant differences were found in clinical or immunological features between patients.	Genetic testing identified primary immunodeficiencies gene defects in almost half of the patients. Extensive genetic screening is recommended for mutations in primary immunodeficiency genes. Direct consequences on patient management due to new monogenic disorders	Identification of CTLA4, LRBA, STAT3, and KRAS mutations in Evans’ syndrome patients Highlighted the need for extensive genetic screening in pediatric- onset of Evans’ syndrome. No distinct features to differentiate Evans’ syndrome patients with or without genetic diagnosis	Genetic testing for primary immunodeficiencies genes in Evans’ syndrome patients is crucial. Extensive genetic screening can guide targeted therapies for Evans’ syndrome patients.	Retrospective study with biased patient recruitment to a reference center Limited number of patients referred for immune deficiencies
Hadjadj *et al.* (2019)	Retrospective cohort study	The study included a national, prospective cohort of 203 patients with early- onset of Evans’ syndrome, with 80 non-selected consecutive individuals undergoing genetic testing.	Genetic testing for pediatric Evans’ syndrome patients with immune gene variants	Sixty-five percent of pediatric Evans’ syndrome cases had genetic determinism. Genetic findings guide treatment choice and have prognostic significance. Sixty-five percent had pathogenic mutations in primary immunodeficiency genes. Twenty-five percent carried probable pathogenic variants in new autoimmune disease genes. Genetic screening is crucial for prognostic and treatment guidance.	Pediatric Evans’ syndrome is genetically determined in at least 65% of the cases. Genetic findings guide targeted treatment choices and have prognostic significance. Systematic genetic screening is recommended in pediatric Evans’ syndrome cases.	Genetic determinism in pediatric Evans’ syndrome Prognostic significance of genetic findings for targeted treatment	Genetic screening guides treatment choices in pediatric Evans’ syndrome. Identifying immune gene variants aids prognosis and targeted treatment selection.	Genetic approach limitations include false-negatives due to selected testing strategies.
Rivalta *et al.* (2019)	Retrospective cross-sectional study	The sample size in the study was 12 pediatric patients affected by Evans’ syndrome referred to the Pediatric Onco-Hematology Unit of Bologna between 2002 and 2018.	Retrospective data collection on pediatric patients with Evans’ syndrome Genetic analysis for mutations in BAFF-R and TACI genes Investigation of TACI mutations in children affected by common variable immunodeficiency	Evans’ syndrome patients had recurrent cytopenia, and underlying immunological or rheumatological diseases. Immunoglobulin replacement therapy was effective in controlling hematological values in patients. Collaborative studies are needed to investigate genetic mutations and pathogenetic mechanisms.	Evans’ syndrome often indicates underlying immunological or rheumatological diseases in children. Collaborative studies are crucial for diagnostic algorithms and therapeutic strategies.	Describes natural history of autoimmune multilineage cytopenias in children.	Early diagnosis crucial for optimal management of immune-mediated cytopenias. Genetic tools aid in identifying mutations associated with primary immunodeficiencies. Collaborative studies are essential for structured diagnostic algorithms and therapeutic strategies.	Lack of international cohort for comprehensive study Limited genetic mutation correlation to clinical pictures
Grimes *et al.* (2021)	Retrospective cross- sectional study	The study involved a cohort of 60 eligible Evans’ syndrome pediatric patients from three tertiary referral care centers during the 6-year study.	Chi-squared and Fisher’s exact tests for categorical data comparison Mann-Whitney U-test for continuous data comparison Kruskal-Wallis ANOVA test for rank comparison in multiple categories	Evans’ syndrome pediatric patients showed excellent outcomes with mTOR inhibitor, sirolimus. Increased identification of immune dysregulation syndromes in children with Evans’ syndrome Mortality rate higher in Evans’ syndrome patients with systemic immune dysregulation	Evans’ syndrome pediatric patients showed excellent outcomes with mTOR inhibitor, sirolimus. Mortality rate was higher in Evans’ syndrome patients with systemic immune dysregulation.	Identification of immune dysregulation disorders in Evans’ syndrome pediatric patients Analysis of diagnostic rates, clinical characteristics, and treatment strategies in Evans’ syndrome Utilization of targeted immune therapies for immune dysregulation disorders	Identifies immune dysregulation in Evans’ syndrome aids to design targeted treatment strategies. Improved diagnostics can guide optimal treatment for Evans’ syndrome pediatric patients.	Low rate of genetic testing due to financial barriers in practice Lack of published clinical data for optimal diagnostic and treatment strategies
Slade *et al.* (2018)	Retrospective cross- sectional study	The study included 179 patients from The Royal Melbourne, Alfred and Austin Hospitals, and the Monash Medical Centre in Victoria, Australia.	Diagnostic approaches for primary antibody deficiencies Infectious and non- infectious manifestations characterized Identification of digenic disease with pathogenic variants in TCF3 and TNFRSF13B	Characterization of 179 patients with primary antibody deficiencies, the majority with common variable immunodeficiency diagnosis Non-infectious complications especially prevailed in common variable immunodeficiency patients. Reduced survival in patients with immune dysregulation and bronchiectasis	Delayed diagnosis of primary antibody deficiencies lead to end-organ damage and complications. Patients with primary antibody deficiencies face increased risk of malignancy and autoimmune diseases. Survival rates are reduced in patients with immune dysregulation. Genetic sequencing accurately aids in diagnosing primary antibody deficiencies, preventing misdiagnoses.	The importance of early and precise diagnosis to prevent severe complications and improve patient outcomes. The genetic underpinnings of predominantly antibody deficiencies, identifying specific mutations associated with these conditions, which contribute to the understanding of the genetic basis of predominantly antibody deficiencies and the potential for genetic testing to aid in diagnosis and management.	Early diagnosis aids in timely treatment and prevents complications. Molecular tests improve diagnostic precision and identify at-risk patients. Improved access to genomic studies enhances diagnostic rates in patients.	Delayed diagnosis leads to end-organ damage like bronchiectasis . Challenges in diagnosing primary antibody deficiencies due to varied clinical presentations Non-infectious manifestations complicate clinical presentation. Risk prediction for non-infectious complications is a major challenge.
Jiang *et al.* (2021)	Prospective study	The study included 44 subjects from an outpatient adult hematology clinic at a tertiary referral center in the United States.	Next-generation sequencing panel with more than 370 genes implicated in inborn errors of immunity Reverse transcription polymerase chain reaction to assess splice site mutation impact Quantitative immunoglobulin testing for autoimmune cytopenias	No cases of inborn errors of immunity were identified in adults with autoimmune thrombocytopenia or Evans syndrome. 18.2% were carriers of pathogenic variants related to inborn errors of immunity, not disease-causing. One TUBB1- related congenital thrombocytopenia case was identified in the study.	No inborn errors of immunity cases were found in adults with autoimmune thrombocytopenia or Evans syndrome. Routine testing for inborn errors of immunity was not recommended for adult patients with autoimmune thrombocytopenia. Genetic testing for inborn errors of immunity in adults returned high number of variants of unknown significance.	The underlying causes of these conditions in adults may differ from those in children, where inborn errors of immunity are more commonly identified in which autoimmune cytopenias are a common manifestation. This contribution is important for understanding the broader implications of inborn errors of immunity in autoimmune conditions and may guide future research and clinical approaches in diagnosing and treating these conditions in adults.	Low diagnostic yield for inborn errors of immunity in adults with autoimmune thrombocytopenia and Evans’ syndrome. Routine testing for inborn errors of immunity is not recommended for adult patients with autoimmune thrombocytopenia.	Lack of inborn errors of immunity identification in adults with autoimmune thrombocytopenia and Evans’ syndrome Low diagnostic yield of genetic testing for inborn errors of immunity in adults with autoimmune cytopenias
Systemic lupus erythematosus								
Hou *et al.* (2023)	Retrospective cohort study	The study included 19 children aged less than 5 years old with systemic lupus erythematosus.	Clinical data review of children with systemic lupus erythematosus under 5 years DNA sequencing of 11 patients to survey genetic etiologies	Early-onset of pediatric systemic lupus erythematosus has distinct clinical features and genetic mutations. Immunological patterns and multisystem organ involvement characterize early-onset of pediatric systemic lupus erythematosus. Renal, mucocutaneous, and hematological systems are commonly affected in pediatric systemic lupus erythematosus.	Early-onset of pediatric systemic lupus erythematosus has an insidious onset with typical symptoms and organ involvement. Genetic testing and immunological screening are crucial for early diagnosis.	Summarized clinical data of 19 children with early-onset of pediatric systemic lupus erythematosus in China. Performed DNA sequencing focusing on systemic lupus erythematosus- associated genes in 11 patients.	Early-onset pediatric systemic lupus erythematosus requires long-term follow-up and early intervention Immunological screening and genetic testing are crucial for early diagnosis. Risk of damage increases overtime, especially in males.	Small sample size minimizes management discrepancies and reflects ethnic homogeneity . Functional tests are needed to clarify gene mutation pathogenicity.
Bhattad *et al.* (2016)	Cross-sectional study	The study had a sample size of 34 children with pediatric-onset systemic lupus erythematosus and 29 age and sex- matched healthy children, enrolled for comparison.	Cross-sectional descriptive study conducted in a pediatric rheumatology clinic Complement system analysis using enzyme- linked immunosorbent assay and nephelometry Genetic mutation analysis and functional assessment of classical complement pathway	Low levels of complement proteins in children with lupus; genetic mutations identified. Complement pathway activity reduced in children with low complement levels Predominant skin manifestations noted in children with decreased complement Clq.	Low complement levels in pediatric-onset systemic lupus erythematosus linked to specific mutations. Mendelian lupus due to C1QA mutations is a significant cause.	First study on inherited complement deficiencies in children with lupus	Identifies early complement deficiencies in pediatric-onset lupus. Highlights distinct clinical profiles in children with inherited complement deficiency. Discusses the importance of C1QA mutations in childhood- onset systemic lupus erythematosus.	Small sample size due to rare pediatric-onset systemic lupus erythematosus Low complement levels due to untested antibodies in some patients
Li *et al.* (2020)	Cross-sectional study	The study enrolled 7 patients with systemic lupus erythematosus with lymphoproliferation from different families, indicating a sample size of 7 participants.	Whole exorne sequencing performed in 7 patients with systemic lupus erythematosus. DNA sequencing and bioinformatic analysis conducted in patient families. Western blot analysis for protein levels in patients with gene mutations	Identified somatic and germline mutations in NRAS, TNFAIP3, and PIK3CD genes. Clinical features include renal, hematologic involvement, recurrent fever, and skin symptoms. Immunologic features show elevated B lymphocyte subgroups and IgG levels.	Somatic NRAS mutation and germline PI3CKD mutation in pediatric systemic lupus erythematosus Need for larger genetic screening in childhood- onset systemic lupus erythematosus for better understanding	Whole exorne sequencing to identify genes associated with systemic lupus erythematosus lymphoproliferation Identification of somatic and germline mutations in NRAS, TNFAIP3, PIK3CD genes Need for gene testing in systemic lupus erythematosus patients with renal and hematologic involvement	Identifies genes NRAS, TNFAIP3, and PIK3CD related with childhood-onset systemic lupus erythematosus. States the need of genetic screening for childhood-onset systemic lupus erythematosus with diverse presentations. Whole genome sequencing can cover limitations of whole- exome sequencing.	Low number of cases, single phenotype, limited healthy controls Whole exorne sequencing limitations in identifying regulatory DNA elements in noncoding regions
Wang *et tal.* (2019)	Case-series study	The research paper involved a sample size of 3 cases in total.	Gene trapping high- throughput sequencing used for gene detection Sanger sequencing for verification of suspicious genes or mutations	Clinical features resembled systemic lupus erythematosus. NRAS mutations found in blood samples of the patients Genetic screening for primary immunodeficiencies recommended in early- onset systemic lupus erythematosus-like patients	RAS-associated autoimmune leukoproliferative disease cases caused by NRAS mutations might be misdiagnosed. Suggested screening of RAS-associated autoimmune leukoproliferative disease in patients with early- onset systemic lupus erythematosus	The study performs DNA sequencing on a subset of patients. The research highlights the typical immunological patterns observed in eo-pSLE, such as ANA positivity and low C3 levels in all children, and emphasizes the importance of immunological screening and genetic testing for early diagnosis.	RAS-associated autoimmune leukoproliferative disease screening for patients with early- onset systemic lupus erythematosus-like and hematologic disorders Genetic testing for primary immunodeficiencies in patients with specific clinical features	No specific limitations were mentioned in the provided context.
Yarbrough *et al.* (2016)	Case-series study		Described a mild, primarily cutaneous phenotype of Aicardi- Goutières syndrome. Recommended testing for SAMHD1 mutations in patients with chilblain lesions.	Identified SAMHD1 mutations in siblings with chilblain lesions Recommended SAMHD1 testing for chilblain lesions and joint stiffness	Aicardi-Goutières syndrome can manifest with cutaneous symptoms, expanding the clinical spectrum. Chilblain lesions can indicate SAMHD1 mutations, requiring neurological evaluation. Early diagnosis of Aicardi- Goutières syndrome in patients with chilblain lesions is crucial.	Described siblings with chilblain lesions leading to genetic testing.	Consider Aicardi- Goutières syndrome in patients with chilblain lesions and unexplained symptoms. SAMHD1 mutations may require brain imaging and intervention. Chilblain lesions can indicate an increased risk for intracranial vasculopathy.	No specific limitations were mentioned in the provided context.
Al-Saud *et al.* (2021)	Case-report study		Case report detailing patient’s presentation, genetic study, and treatment plan Preliminary immunological workup and genetic study for purine nucleoside phosphorylase deficiency	The patient had purine nucleoside phosphorylase deficiency, systemic lupus erythematosus, lymphoma, and recurrent infections. Genetic study revealed homozygous missense mutation in the PA/Pgene. The patient had severe lymphopenia and low lymphocyte proliferation. Uric acid levels were normal after blood transfusion. The patient passed away before hematopoietic stem cell transplantation.	Challenges in recognizing low lymphocyte proliferation, deficiency in systemic lupus erythematosus patients Evaluation for primary immunodeficiencies in children with autoimmune diseases Heterogeneity of clinical syndrome associated with PNP deficiency	Rare co-occurrence of systemic lupus erythematosus and purine nucleoside phosphorylase deficiency with immune deficiency Challenges in recognizing purine nucleoside phosphorylase deficiency in systemic lupus erythematosus patients	Highlighted challenges in diagnosing purine nucleoside phosphorylase deficiency in systemic lupus erythematosus patients. Emphasized the need for primary immunodeficiency evaluation in autoimmune diseases. Demonstrated the clinical heterogeneity associated with purine nucleoside phosphorylase deficiency.	Challenges in recognizing low lymphocyte proliferation, deficiency in systemic lupus erythematosus patients Complexity and heterogeneity of clinical syndrome associated with purine nucleoside phosphorylase deficiency
Inflammatory bowel disease								
Crowley *et al.* (2020)	Cross-sectional study	The study identified 40 rare variants associated with 21 monogenic genes among 31 children with inflammatory bowel disease, indicating a sample size of over 1,000 participants.	Whole exorne sequencing used to determine monogenic inflammatory bowel disease gene prevalence	Identified 40 rare variants in 21 monogenic genes among 31 children with inflammatory bowel disease. Characterized monogenic inflammatory bowel disease features: abdominal pain, loose stool, vomiting, weight loss. Monogenic inflammatory bowel disease was associated with early onset, family history, and surgery. 3% of the children had rare variants in genes linked to pediatric inflammatory bowel disease. One of the patients had variants correctable with allogeneic hematopoietic stem cell transplantation.	Rare variants were found in monogenic genes related to pediatric inflammatory bowel disease. Genetic factors influence prognosis and personalized therapy selection. Monogenic inflammatory bowel disease is rare but crucial in pediatric inflammatory bowel disease analysis.	Identified rare variants in monogenic genes associated with pediatric inflammatory bowel disease. Provided insights into genetic factors, prognosis, and therapy selection.	Genetic factors influence prognosis and therapy selection in pediatric inflammatory bowel disease. Monogenic inflammatory bowel disease diagnosis is crucial for personalized treatment strategies. Allogeneic hematopoietic stem cell transplantation can correct gene variants in some monogenic- derived inflammatory bowel disease.	Whole genome sequencing limitations include poor exon coverage and non-coding regions. Functional testing absence affects genetic interpretation for therapeutic decisions. Rapidly increasing gene discovery related to monogenic inflammatory bowel disease may underestimate their contribution.
Kelsen *et al.* (2016)	Cross-sectional study	The study included DNA analysis from 125 patients with very early-onset inflammatory bowel disease and 19 parents, 4 of them also with inflammatory bowel disease. Additionally, 210 exorne samples were obtained from patients with pediatric inflammatory bowel disease (n = 45), adult-onset Crohn’s disease (n = 20), and healthy controls (n = 145) for comparison.	Exorne capture by Agilent SureSelect V4 and sequencing by lllumina HiSeq. Patients were recruited from Children’s Hospital of Philadelphia and University of Kiel. Analysis of DNA from 125 patients with very early-onset inflammatory bowel disease and 19 parents Variant calling with minor allele frequency inferior to 0.1% and scaled Combined annotation-dependent depletion (CADD) scores inferior to 10. Focus on genes linked to primary immunodeficiencies and related pathways.	Identified rare variants in primary immunodeficiency- related genes in very early- onset inflammatory bowel disease patients. An average of 4.14 variants per child were identified in the very early- onset inflammatory bowel disease cohort.	Identified variants in genes regulating B and T cell functions Potential novel variants in CD19, MSH5, and IL10RA genes Enrichment of variants in primary immunodeficiency genes in very early-onset inflammatory bowel disease Link between T cell deficiency and B cell function impairment	Detection of rare variants in primary immunodeficiency genes using whole genome sequencing Identification of novel variants in genes regulating B and T cell functions	Identifying rare variants in primary immunodeficiency genes in very early onset inflammatory bowel disease Potential for discovering novel inflammatory bowel disease-associated variants through exorne sequencing Enrichment of variants in primary immunodeficiency genes associated with very early-onset inflammatory bowel disease	Limited sample size focused on primary immunodeficiency pathways Variants may not affect gene function as determined in analysis.
Fang *et al.* (2018)	Retrospectivecross- sectional study	The study involved 16 patients who underwent whole genome sequencing or targeted gene panel sequencing: six patients were tested by whole genome sequencing and 12 by targeted gene panel sequencing. Additionally, 2 patients were tested by both (whole genome sequencing and targeted gene panel sequencing).	Retrospective analysis of children with very early-onset inflammatory bowel disease using genetic sequencing Whole exorne sequencing and target gene panel sequencing for genetic analysis	Monogenic inflammatory bowel disease patients had younger diagnosis age than the non-monogenic group. Higher incidence of perianal disease in monogenic inflammatory bowel disease group IL10RA mutation was predominant in the monogenic inflammatory bowel disease cohort. Monogenic and non- monogenic inflammatory bowel disease showed similar clinical features.	Monogenic inflammatory bowel disease was prevalent in very early-onset inflammatory bowel disease cohort. IL10RA mutation was the most common. Clinical similarities between monogenic and non-monogenic inflammatory bowel disease. Next-generation sequencing vital for diagnosing monogenic inflammatory bowel disease	The paper explores the genotypic aspects of inflammatory bowel disease in these young children. This involves examining genetic markers or mutations that may be associated with the disease, contributing to a better understanding of its genetic basis in the pediatric population.	High proportion of monogenic inflammatory bowel disease in the very early-onset group Next generation sequencing crucial for diagnosing monogenic inflammatory bowel disease	No functional analyses of novel mutations performed, requiring confirmation studies Not all very early- onset inflammatory bowel disease patients underwent genetic testing, affecting cohort representation.
Ashton *et al.* (2020)	Retrospective cross-sectional study	The study included 401 patients in the analysis making this the sample size for the research.	The patients were recruited from pediatric inflammatory bowel disease service at Southampton Children’s Hospital. Whole exorne sequencing performed on pediatric-onset inflammatory bowel disease patients. Genetic variants assessed against phenotype at diagnosis and follow-up.	11.5% percent had monogenic variants; 7.3% had A/OD2-related disease. Identification of pathogenic variants in 68 monogenic inflammatory bowel disease genes Median age at diagnosis was 11.92 years. Recurrent variants observed in NOD2, TRIM22, CD40LG. and others. Personalized therapy based on NOD2 status for prognosis and intervention.	Identified monogenic variants in pediatric inflammatory bowel disease patients with distinct clinical phenotypes- NOD2 status indicates risk of stricturing disease and surgery. Novel variants in TRIM22, CD40LG, WAS, and NCF2 genes Compound heterozygous variants confirmed in DCLRE1C, NCF2, TRIM22, and NOD2.	Improved predictive algorithms using genetic diagnoses and exorne sequencing data. Identified clinically relevant variants within monogenic inflammatory bowel disease genes.	Genetic sequencing aids in designing personalized therapy in pediatric inflammatory bowel disease patients. NOD2 status guides prognosis and intervention for stricturing disease. Rare monogenic variants contribute to disease etiology in pediatric inflammatory bowel disease. Missing heritability in inflammatory bowel disease may be due to rare mutations.	Functional validation of novel variants remains challenging without confirmation. Imperfection of deleteriousness metrics limits GenePy score for pathogenicity assessment.
Lega *et al.* (2020)	Cohort study	The sample size was a total of 93 patients diagnosed with very early-onset inflammatory bowel disease and early onset-inflammatory bowel disease with severe/atypical phenotypes who underwent a genetic workup. Out of the 93 patients, 47 underwent candidate gene sequencing, and 84 patients underwent next generation sequencing.	Candidate-gene sequencing guided by clinical features for known monogenic diseases Next generation sequencing for finding new causative genetic variants Targeted gene panel sequencing and whole exorne sequencing	Ninety-three patients included, 12 reached a genetic diagnosis. Genetic diagnosis impacted management of 11 patients (92%). Next generation sequencing revealed 12 cases of monogenic inflammatory bowel disease.	Genetic diagnosis impacts patient management in suspected monogenic inflammatory bowel disease. Early onset, extraintestinal manifestations, and male sex suggest genetic defects.	Shift from candidate- gene to next generation sequencing for monogenic inflammatory bowel disease diagnosis Impact of genetic diagnosis on patient management	Genetic diagnosis impacts patient management in suspected monogenic inflammatory bowel disease. Next generation sequencing is preferred for patients with nonspecific phenotypes. Candidate-gene sequencing guided by clinical features specific for known monogenic diseases. - Early age at disease onset and extraintestinal manifestations suggest genetic defects.	Retrospective data collection may affect data quality. Potential selection bias due to multiple participating centers
Ziv *et al.* (2020)	Case-report study		Whole-exome sequencing for genetic analysis Sanger sequencing for mutation confirmation Next generation sequencing for immune repertoire analysis Telomere length evaluation by in-gel hybridization assay Mass cytometry on peripheral blood mononuclear cells for immune analysis	RTEL1 mutation linked to infantile-onset inflammatory bowel disease with severe immunodeficiency Patient had unique clinical features, severe ulcerative colitis, and immunodeficiency. Immune landscape alterations, short telomeres, and polyclonal T and B cells	RTEL1 mutations linked to immune alterations and infantile onset inflammatory bowel disease High index of suspicion needed in Ashkenazi Jewish families.	Unusual clinical course of infantile- onset colitis due to RTEL1 mutation Genetic, molecular, and immunological workup of the patient	RTEL1 mutations linked to infantile-onset inflammatory bowel disease in Ashkenazi Jewish families High index of suspicion needed for early diagnosis in affected families.	Limited data on intestinal inflammation in patients with RTEL1 mutation Unclear if patients with telomere disorders develop intestinal inflammation
Malik *et al.* (2021)	Case-report study		Whole exorne sequencing analysis performed to identify gene variants Extensive immunology I workup conducted to rule out primary immunodeficiencies	Laboratory tests showed low Immunoglobulin levels and normal albumin levels. Patient had preserved protective antibody titers to various vaccine antigens. Immunology workup ruled out other primary immunodeficiencies and complement deficiencies. Patient had recurrent bronchitis, ear infections, and zoster post-chickenpox. Family history included psoriasis, psoriatic arthritis, and autoimmune thyroiditis.	Genetic variants contribute to inflammatory bowel disease-like intestinal inflammation. Early diagnosis is crucial for managing multi-system autoimmune inflammatory diseases.	Genetic variants in inborn errors of immunity causing autoimmune manifestations and chronic inflammation Diagnosis and treatment of multisystem autoimmune inflammatory disease Immunolaboratory investigation results for the patient’s immune system	Identifying monogenic disorders inflammatory bowel disease-like intestinal inflammation Highlighting the importance of genetic variants in autoimmune inflammatory diseases	Limited exclusion of Crohn’s disease due to granuloma presence Patient intolerance to methotrexate for arthritis and inflammatory bowel disease treatment.
Vardi *et al.*(2020)	Case-report study		Whole exorne sequencing for genetic analysis Mass cytometry and flow cytometry for immunophenotyping Next generation sequencing for immune repertoire analysis	*LRBA* deficiency showed marked immunological changes in innate and adaptive cells. Reduced cytokine production of T cells in *LRBA*-deficient patients	LRBA deficiency causes significant immunological changes in innate and adaptive cells. Genetic studies are crucial for unique phenotypes regardless of age.	Therapeutic implications of monogenic disorders in adult patients Importance of advanced genetic studies in patients with unique phenotypes	Advanced genetic studies benefit unique clinical phenotypes, improving patient care. Next generation sequencing aids in diagnosing monogenic disorders in adult patients.	Limited data on hematopoietic stem cell transplantation success rates in adult primary immunodeficiencies. Challenges in I genotype-phenotype correlation in LRBA deficiency patients
Edwards *et al.* (2023)	Case-report study		Whole exorne sequencing of genomic DNA for genetic analysis Flow cytometry detection of phosphorylated ribosomal protein S6 for signaling evaluation Collection of medical information and blood samples for diagnostic workup	Identification of biallelic missense variants in *LCP2* affecting SLP76 protein . Reduced SLP76 expression in B, T, and NK cells. Impaired neutrophil function, normal B and T cell numbers Decreased intracellular SLP76 protein levels In the patient’s immune cells. Defects in antigen receptor signaling due to *LCP2* variants	Biallelic LCP2 variants impair immune signaling, causing combined immunodeficiency. Defects in SLP76 and PI3K signaling lead to early immune dysregulation. Platelet defects are absent in patients with SLP76variants.	First case of compound heterozygous LCP2 variants in a young adult Impaired PI3K signaling in a patient with biallelic *LCP2* variants	Identifies *LCP2* variants causing combined mmunodeficiency with immune dysregulation. Provides insights into impaired PI3K signaling and immune defects. Offers a rapid ex vivo functional test for antigen receptor signaling defects. Demonstrates the impact of *SLP76* variants on immune signaling pathways.	Limited sample size for comprehensive generalization Lack of detailed discussion on long-term patient outcomes
Olyha *et al.* (2023)	Case-series study	The research paper involved an international cohort of 14 patients with deficiency in ELF4, X-linked which represents the sample size of participants in the study.	Unpaired t tests for luciferase assays and cytometry by time-of- flight (CyTOF^®^) analysis DNA extraction, sequencing, alignment, variant identification, and Sanger sequencing	Patients with deficiency in *ELF4* (X-linked) showed heterogeneous clinical phenotype with various inflammatory symptoms. Biologies targeting IL-1, IL-12p40, and TNF-a are effective treatments. Skin inflammation, oral ulcers, arthritis, and gastrointestinal symptoms are common.	Patients with deficiency in ELF4 (X-linked) exhibit diverse clinical symptoms and inflammatory markers. Biologic products targeting IL-1, IL-12p40, and TNF-a showed efficacy. Skin inflammation varies among the patients with deficiency in ELF4 (X-linked) during symptomatic flares.	Described a cohort of patients with deficiency in ELF4 (X-linked) with different clinical phenotypes. Highlighted immunological characteristics, histopathological findings, and therapeutic interventions of patients with deficiency in ELF4.	Identifying *ELF4* deficiency aids targeted therapeutic interventions for autoinflammatory syndromes. Patients with deficiency in *ELF4* (X-linked) benefit from anti-inflammatory agents and biologic products targeting TNF-a. *ELF4* variants contribute to inflammation in patients with deficiency in ELF4, X-linked.	Heterogeneity in immune populations may affect functional responses in patients with deficiency in *ELF4*, X-linked. Not all deficiencies in *ELF4* induce gastrointestinal symptoms or have family history.

### Assessment of methodological quality

**Figure 2.**
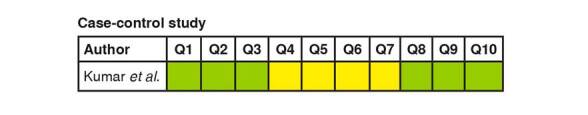
Individual risk of bias analysis of a case-control study **Questions:** Green: low risk of bias; Yellow: unclear risk of bias; Red: high risk of bias Q1. Were the groups comparable other than the presence of disease in cases or the absence of disease in controls? Q2. Were cases and controls matched appropriately? Q3. Were the same criteria used for identification of cases and controls? Q4. Was exposure measured in a standard, valid and reliable way? Q5. Was exposure measured in the same way for cases and controls? Q6. Were confounding factors identified? Q7. Were strategies to deal with confounding factors stated? Q8. Were outcomes assessed in a standard, valid and reliable way for cases and controls? Q9. Was the exposure period of interest long enough to be meaningful? Q10. Wasappropriate statistical analysis used?

**Figure 3.**
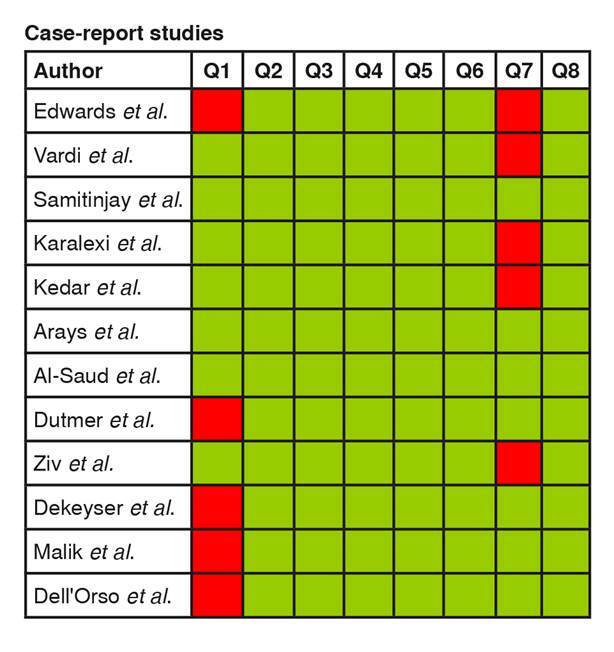
Individual risk of bias analysis of case-report studies **Questions:** Green: low risk of bias; Yellow: unclear risk of bias; Red: high risk of bias Q1. Were patient’s demographic characteristics clearly described? Q2. Was the patient’s history clearly described and presented as a timeline? Q3. Was the current clinical condition of the patient in the presentation clearly described? Q4. Were diagnostic tests or assessment methods and the results clearly described? Q5. Was the intervention(s) or treatment procedure(s) clearly described? Q6. Was the post-intervention clinical condition clearly described? Q7. Were adverse events (harms) or unanticipated events identified and described? Q8. Doesthe case report provide takeaway lessons?

**Figure 4.**
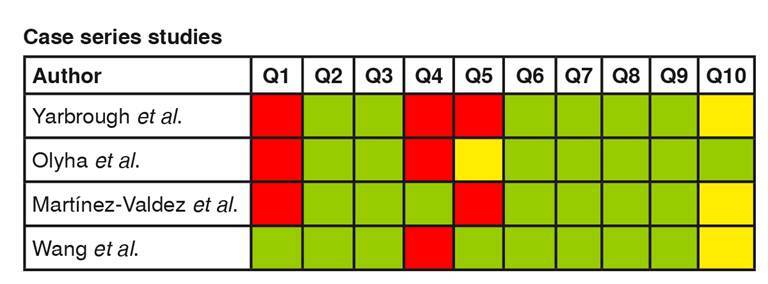
Individual risk of bias analysis of case-series studies **Questions:** Green: low risk of bias; Yellow: unclear risk of bias; Red: high risk of bias Q1. Were there clear criteria for inclusion in the case series? Q2. Was the condition measured in a standard, reliable way for all participants included in the case series? Q3. Were valid methods used for identification of the condition for all participants included in the case series? Q4. Did the case series have consecutive inclusion of participants? Q5. Did the case series have complete inclusion of participants? Q6. Was there clear reporting of the demographics of the participants in the study? Q7. Was there clear reporting of clinical information of the participants? Q8. Were the outcomes or follow-up results of cases clearly reported? Q9. Was there clear reporting of the presenting site(s)/clinic(s) demographic information? Q10. Wasstatistical analysis appropriate?

**Figure 5.**
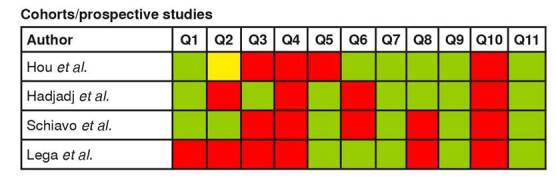
Individual risk of bias analysis of cohort/prospective studies **Questions:** Green: low risk of bias; Yellow: unclear risk of bias; Red: high risk of bias Q1. Were the two groups similar and recruited from the same population? Q2. Were the exposures measured similarly to assign people to both exposed and unexposed groups? Q3. Was the exposure measured in a valid and reliable way? Q4. Were confounding factors identified? Q5. Were strategies to deal with confounding factors stated? Q6. Were the groups/participants free of the outcome at the start of the study (or at the moment of exposure)? Q7. Were the outcomes measured in a valid and reliable way? Q8. Was the follow up time reported and sufficient to be long enough for outcomes to occur? Q9. Was follow up complete, and if not, were the reasons to loss to follow up described and explored? Q10. Were strategies to address incomplete follow up utilized? Q11. Wasappropriate statistical analysis used?

**Figure 6.**
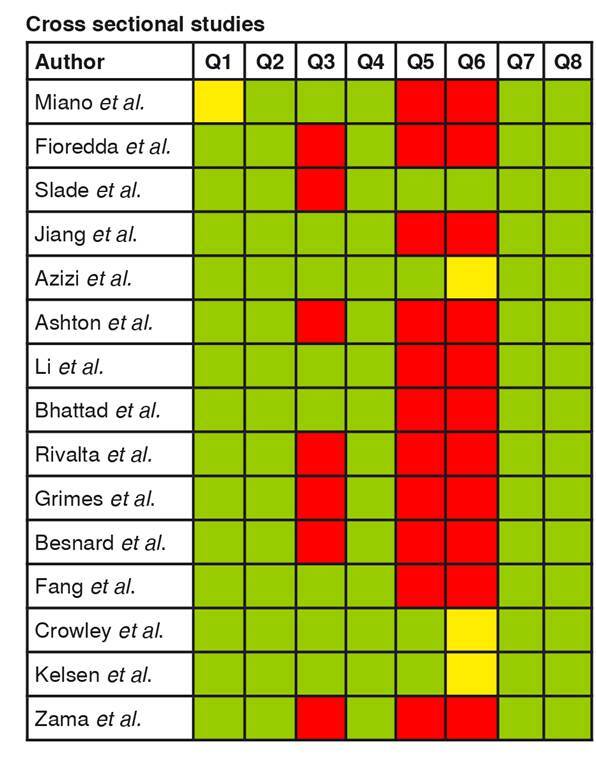
Individual risk of bias analysis of cross-sectional studies **Questions:** Green: low risk of bias; Yellow: unclear risk of bias; Red: high risk of bias Q1. Were the criteria for inclusion in the sample clearly defined? Q2. Were the study subjects and the setting described in detail? Q3. Was the exposure measured in a valid and reliable way? Q4. Were objective, standard criteria used for measurement of the condition? Q5. Were confounding factors identified? Q6. Were strategies to deal with confounding factors stated? Q7. Were the outcomes measured in a valid and reliable way? Q8. Wasappropriate statistical analysis used?

After the selection process, 36 articles were included and assessed for methodological quality using the Joanna Briggs Institute critical appraisal checklist for individual risk of bias analysis ^7^^,^^8^. The manuscripts were sorted by type of study: case-control (n = 1), case report (n = 12), case series (n =4), cohort/prospective (n = 4), and cross-sectional (n = 15) (figures 2-6).

### Cytopenias and Inborn errors of immunity In adult and pediatric populations

We found 20 studies on pediatric and some adult populations, providing a comprehensive view of how certain conditions, such as autoimmune cytopenias, correlate with underlying genetic defects. The most reported cytopenias include Evans’ syndrome, autoimmune hemolytic anemia, autoimmune thrombocytopenia, and autoimmune neutropenia.

Case-control study

Case-report studies

Case series studies

Cohorts/prospective studies

Cross sectional studies

The Italian neutropenia registry data included 79 children with late-onset neutropenia. These types of neutropenia showed a lower rate of resolution, B and natural killer (NK) lymphopenias, and an increased need for colony- stimulating factor ^9^. Despite low B and NK cell counts, they did not study inborn errors of immunity in those patients ^9^.

A 35-year Italian study on Evans’ syndrome describes a male preponderance (23/40) with concomitant Evans’ syndrome in 18/40 patients; 9/40 had a family history of autoimmunity, and 24/40 (60%) presented lymphoproliferation and multiple positive autoantibodies (ENA, ANCA, ASMA) ^10^. In this group, 18/40 patients had an underlying genetic defect in *LIG4, ADA2, CARD11, IKBGK, STAT3, CTLA4, LRBA, TNFSF13B, CASP1O*, and FAS ^10^. Kumar *et al*. analyzed the immune profile of 24 pediatric Evans’ syndrome patients. The median age was 12 (range: 1 to 19 years), 17 patients had lymphoproliferation, and 3 had pulmonary and gastrointestinal manifestations, decreased naive CD4+ T cells, and class-switched memory B cells. The most frequent genetic mutations were in *CTLA4* and *LRBA*^11^.

Refractoriness and persistence of cytopenias are common. One case involved a misdiagnosis of autoimmune thrombocytopenia in a child with microthrombocytopenia, immune deficiency, and severe eczema who had Wiskott-Aldrich syndrome. This scenario highlights the need to consider alternative diagnoses like inborn errors of immunity in persistent and refractory cytopenias ^12^. Another report describes a female patient from consanguineous parents with severe refractory hemolytic anemia and recurrent viral and bacterial infections. The whole exorne sequencing revealed a homozygous mutation in the *LRBA* gene ^13^ (table 2).

**Table 2.** Red flags to suspect inborn errors of immunity in patients with autoimmune diseases

Autoimmune disease	Red flags to suspect inborn errors of immunity	
Autoimmune cytopenias	1.	Chronic, recurrent and refractory cytopenias
	2.	Recurrent Infections
	3.	Poly-autoimmunity (autoimmune hemolytic anemia, autoimmune thrombocytopenia, autoimmune neutropenia) along with other findings, such as celiac disease, thyroiditis, and psoriasis.
	4.	Chronic lymphoproliferation (hepatomegaly, splenomegaly, or lymphadenopathy)
	5.	Severe infectious complications associated with immunosuppressive treatment
Systemic lupus erythematosus	1.	Age of onset before five years
	2.	Atypical clinical manifestations of the disease
	3.	Severe organ involvement
	4.	Chronic lymphoproliferation
	5.	Refractory immune-mediated cytopenias
	6.	Recurrent infections
	7.	Chilblain lesions
	8.	Resistance to conventional therapies
Inflammatory bowel disease	1.	Personal history of recurrent infections
	2.	Resistance to conventional therapies
	3.	Atypical symptoms of inflammatory bowel disease
	4.	Multiorgan involvement associated with manifestations of poly-autoimmunity
	5.	Abnormal findings in immunological workup
	6.	Atypical endoscopic or histological findings
	7.	Young age at presentation
	8.	Family history of neoplasms and autoimmune manifestations
	9.	Consanguineous parents
	10.	Higher rates of severe perianal/fistulating disease*
	11.	Need for surgical intervention*

* Red flag seen mainly in inflammatory bowel disease pediatric patients

These findings align with other studies. The retrospective registry of Italy evaluated autoimmune hemolytic anemia, autoimmune thrombocytopenia, and autoimmune neutropenia in 47 patients aged 0 to 18. They had a mean diagnosis age of 15.2 years and a peak cytopenia age of approximately 8.6 years. By gene panel sequencing, they found that 19/47 patients had inborn errors of immunity, with common variable immunodeficiency being the most frequent (47%), followed by unclassified disorders (26%) and autoimmune lymphoproliferative syndrome (21%). Autoimmune thrombocytopenia was the most frequent cytopenia (81%), followed by autoimmune hemolytic anemia (23%), autoimmune neutropenia, and Evans’ syndrome (21%). The immunophenotyping identified T/B lymphopenia, decreased naive T cells, switched memory B cells, plasmablasts, and immunoglobulins with statistical significance ^14^.

The Immunodeficiency Research Center retrospective cohort at the Children’s Medical Center in Iran evaluated 471 patients with primary antibody deficiencies, of which 67.3% were male. Autoimmune disorders, as the first presentation of inborn errors of immunity, were reported in 2.5% of the patients. Autoimmune thrombocytopenia, autoimmune hemolytic anemia, and type I diabetes mellitus were the most common. Patients with primary antibody deficiencies and autoimmunity had fewer counts of CD3+ and CD4+ T cells than those without autoimmunity. The number of CD19+ B cells was higher in patients with primary antibody deficiencies and autoimmunity. Among patients with inborn errors of immunity and autoimmune diseases, 87/125 had one autoimmune disease, while 38/125 (30.4%) had polyautoimmunity. The Treg count was lower in patients with primary antibody deficiencies and autoimmunity ^15^.

Schiavo *et al.* performed a prospective analysis of 30 patients with refractory cytopenias requiring second and third-line management. They used a next-generation sequencing panel with samples of 14/21 patients and whole exorne sequencing for 7/21 individuals. They found variants of interest in *FAS, ST AT3* (gain-of-function), *UNC13D, PI3KD, KMT2D, CARD11*, and AIRE genes ^16^.

In some patients, autoimmune cytopenias were complicated by infectious diseases. Samitinjay *et al.* reported a late-stage adolescent with severe autoimmune hemolytic anemia and malaria infection; her medical history revealed recurrent upper and lower respiratory tract infections and hypogammaglobulinemia, resulting in a final diagnosis of common variable immunodeficiency ^17^. A 13-month-old patient with previous varicella zoster vaccination was admitted with Evans’ syndrome. Treatment with corticosteroids favored the dissemination of the infection, leading to a fatal outcome. Genetic sequencing revealed a hypomorphic mutation in *RAG2* (loss-of-function) ^18^. Similar manifestations have been documented in adulthood, such as a woman with a medical history of autoimmune thrombocytopenia diagnosed at 40 years, refractory to steroid treatment, requiring splenectomy at 42 years, followed by rituximab. She had an abnormal vaccine response to protein antigens despite having been vaccinated before the splenectomy. Reduced immunoglobulin levels lead to a diagnosis of common variable immunodeficiency ^19^.

In some cases, the relationship between inborn errors of immunity and autoimmunity appears through other manifestations, such as neurologic compromise. A 49-year-old patient had splenomegaly, lymphadenopathy, and recurrent autoimmune cytopenias since the age of 15. He received corticosteroids, intravenous immunoglobulins, romiplostim injections, and rituximab. He presented with abnormal right plantar reflex, positive for the Romberg test, ataxia, and lateral pulsion in gait. Analysis showed severe panhypogammaglobulinemia, increased vitamin B12 levels, elevated TCR a(B+, and double-negative T lymphocytes (CD4-/CD8-). The whole exorne sequencing study demonstrated a heterozygous missense variant in the FAS gene, resulting in a final diagnosis of autoimmune lymphoproliferative syndrome ^20^. Dell’Orso *et al.* reported a female who suffered from autoimmune hemolytic anemia, autoimmune thrombocytopenia, and chronic lymphoproliferation treated with high-dose steroid therapy, anti-thymocyte globulin transfusions, and cyclosporine A. The autoimmune hemolytic anemia was severe and associated with myelodysplastic syndrome and elevated double-negative T lymphocytes, leading to a diagnosis of autoimmune lymphoproliferative syndrome. A next-generation sequencing panel identified two germline mutations in the *ADA2/CECR1* gene ^21^.

In the pediatric population and refractory cytopenias, we find the registry of 12 patients diagnosed with Evans’ syndrome in the pediatric population with refractory cytopenias. The mean age of cytopenia onset was 9 years, and the average time to diagnosis was 7.4 years. Five of nine patients had a final diagnosis of an inborn error of immunity. Common variable immunodeficiency was the most frequent, followed by autoimmune lymphoproliferative syndrome and Rubinstein-Taybi syndrome ^22^. Other retrospective and prospective studies of pediatric Evans’ syndrome included reports of celiac disease, vitiligo, myositis, glomerulonephritis, type I diabetes, autoimmune hepatitis, pericarditis, and eczema ^23^.

Clinical findings such as inflammatory bowel diseases, hepatosplenomegaly, lymphadenopathy, and antibody positivity -such as ANA, ENA, ASMA, ANCA, and anti-dsDNA- have been documented in the previously mentioned populations. Sequencing in these patients found a high frequency of mutations in genes already reported in previous studies during this review, such as *LRBA, CTLA4, STAT3, RAG1*, and *TNFRSF6*, and others like *KRAS, PIK3CD, CBL, ADAR1*^24^. Pediatric Evans’ syndrome is associated with a high frequency of potentially harmful variants in immune genes. This is associated with greater disease severity ^25^. Additionally, there is a marked tendency toward refractoriness to treatment and disease severity ^26^.

Adult registries are less frequent. In 2018, Slade *et al*. conducted a cross- sectional clinical analysis of 179 patients with primary antibody deficiencies managed in Australia, 98 of whom were women. The median diagnosis delay was 9 years. Common variable immunodeficiency was the most frequent diagnosis, with 4/116 having NF-k/31 deficiency and 3/116 heterozygous for TNFSRF13B. Common variable immunodeficiency patients developed cytopenia, enteropathy, splenomegaly, lymphadenopathy, and granulomatous infiltration more frequently, often as the first manifestation of inborn errors of immunity ^27^. Jiang *et al.* performed a prospective study to determine the prevalence of inborn errors of immunity in a group of patients with chronic autoimmune thrombocytopenia and Evans’ syndrome. They enrolled 49 patients, with a mean age of 49 years, 56.8% of whom were women. They performed a next generation sequencing panel. No cases of inborn errors of immunity were identified despite the presence of subjects with a personal history of autoimmunity (45%) and disease onset before 40 years ^28^ (table 2).

### Systemic lupus erythematosus

Systemic lupus erythematosus is a heterogeneous inflammatory and autoimmune disease with diverse phenotypes and clinical courses. Between 15 and 20% of systemic lupus erythematosus appears before 18 years and is known as pediatric or early-onset systemic lupus erythematosus; when it appears before five years of age, it is known as very early-onset. It usually has atypical clinical manifestations, greater organ involvement, and poor prognosis ^29^. Most early-onset systemic lupus erythematosus cases are frequently associated with monogenic variants that lead to severe immune dysregulation. In the pediatric population, we reviewed six articles: four retrospective observational studies, one case report, and one case series.

Hou *et al.* described a cohort of 339 patients in China, selecting those with an age of presentation younger than five years, and performed whole exorne sequencing. Nineteen patients presented with the disease before the age of five, 13 were females, and 11 underwent genetic studies. The mean age of onset was 3.7 years; the diagnosis delay was longer in male patients (9 months). Four out of 19 patients had a systemic lupus erythematosus family history, 75% in first-degree relatives. The most prevalent clinical characteristics were renal involvement (94.74%), mucocutaneous (94.74%), hematological (89.47%), respiratory (89.47%), digestive (84.21%), cardiovascular (57.89%), and neuropsychiatric (52.63%) compromise. Low C3 complement and positive ANA antibodies were identified in all children. Remarkably, four patients developed overlapping clinical diseases with systemic sclerosis, juvenile idiopathic arthritis, and psoriasis. Authors identified 13 variants in 81.82% of the patients in the genes TREX1, *PIK3CD, LRBA, KRAS, STAT4, C3, ITGAM, CYBB, TLR5, RIPK1, BACH2, CFHR5*, and *SYK*^29^. This study concluded that early-onset systemic lupus erythematosus has an insidious and atypical onset and often presents with severe organ involvement ^29^.

Bhattad *et al*. described 34 patients with early-onset systemic lupus erythematosus. Those with complement deficiency underwent a whole exorne sequencing study. Complement levels were low in 7/34; patients with low Clq had a specific phenotype with an early age at onset, low dsDNA antibody titers, and prominent skin manifestations. The genetic analysis showed relevant variants in 3/7 patients in the *C1qA* and properdin (*CFP*) genes ^30^.

Li *et al*. performed whole exorne sequencing in seven Chinese patients with juvenile systemic lupus erythematosus and chronic non-malignant lymphoproliferation. The average age of onset was 5 years, with hematuria or proteinuria in 7/7, cytopenias in 6/7, and recurrent fever in 6/7. They describe genetic variants in 6/7 patients; 4/7 patients had activating somatic de novo variants in the NRAS gene, one patient had a PIK3CD variant, and one had a TNFAIP3 mutation ^31^.

Wang et al. reported three patients with systemic lupus erythematosus and somatic mutations in the NRAS gene; their main clinical manifestations were immune-mediated thrombocytopenia, polyarthritis, positive autoantibodies, persistent monocytosis, and hepatosplenomegaly ^32^. Yarbrough *et al*. reported the case of three brothers with chilblains, with only one of them having neurologic compromise. They presented Raynaud’s syndrome and chronic polyarticular arthritis. The older brother had white matter calcifications in the basal ganglia but no circulating autoantibodies. The genetic study showed heterozygosity in SAMHD1, confirming an Aicardi-Goutieres syndrome ^33^.

Al-Saud *et al.* reported a previously healthy 7-year-old girl with pediatric systemic lupus erythematosus poorly controlled with immunosuppressive therapy. She had severe cytopenias, required multiple hospitalizations due to severe respiratory infections, and had recurrent varicella zoster infection. With the suspicion of an inborn error of immunity, she underwent a whole exorne sequencing study, revealing a purine nucleoside phosphorylase deficiency ^34^ (table 2).

### Inflammatory bowel disease

Inflammatory bowel disease, comprised of ulcerative colitis, Crohn’s disease, and unclassified inflammatory bowel diseases, are chronic conditions of the gastrointestinal tract affecting children and adults ^35^. Most patients with monogenic inflammatory bowel disease will have symptoms early in childhood. However, some will not be diagnosed until adulthood. We reviewed ten articles: four case reports, one case series, and five observational studies. Only two articles were about monogenic inflammatory bowel disease in adults, and the remaining eight were about the pediatric population.

Crowley *et al*. performed whole exorne sequencing analyses in a cohort of 1,005 children with inflammatory bowel disease between 0-18 years old in Canada and their family members (2,305 samples total, 1,005 pediatric patients, and 1,300 parents and siblings) ^35^. The patients had a 1.5:1 male-to-female ratio; 40% were diagnosed with ulcerative colitis/unclassified inflammatory bowel disease and 60% with Crohn’s disease. The median age of diagnosis was 11.6 years, and the median age at symptom onset was 10.6 years ^35^. Kelsen *et al.* analyzed 125 children less than five years old having very early onset inflammatory bowel diseases. The onset age in most children (84%) was under two years old. Consanguinity was frequent, with a history of 10% first-degree and 18% second-degree relatives ^36^. Fang *et al.* studied 54 Chinese children with very early-onset inflammatory bowel disease; 37 were males, and 17 were females. Of the 54 patients, 31 (57%) had disease onset before two years old ^37^. In the monogenic Canadian inflammatory bowel disease group, 14 (45%) were diagnosed with ulcerative colitis/unclassified inflammatory bowel disease, and 17 (55%) with Crohn’s disease ^35^; in the Chinese group, 72.2% of patients were diagnosed with Crohn’s disease, and 27.8% with ulcerative colitis/unclassified inflammatory bowel disease ^37^. The features significantly associated with monogenic inflammatory bowel disease compared with the remaining cohort were the onset age of the disease (< 2 years), extraintestinal manifestations, family history of autoimmune disease, and surgery ^35^. Similarly, Kelsen *et al*. described severe disease activity in 61% of the patients. Surgical interventions were frequent: 14 patients had undergone colectomy, two had perianal disease, and 15 had an ileostomy ^36^.

After the genetic analysis, Crowley *et al*. found 40 pathogenic variants in 21 genes of 31/1,005 patients. These variants were validated using Sanger sequencing. The most frequent variants were in *XIAP* (5/31,16%), *DOCKS* (3/31,10%), *LRBA* (2/31,6%), *GUCY2C* (2/31,6%), *FOXP3* (2/31,6%), and *ARPC1B* (2/31,6%) ^35^. Nine of the 54 Chinese patients were diagnosed with monogenic inflammatory bowel disease, 5/9 had a pathogenic variant in IL10R, 2/9 were diagnosed with chronic granulomatous disease with *CYBB* variants, one patient had *XIAP* deficiency, and one had a *TNFRSF13B* mutation ^37^. The frequency of monogenic variants in the Canadian very early-onset inflammatory bowel disease study was 7.8%. Unexpectedly, they found a prevalence of monogenic etiology in 2.3% of children aged six years and older. In this cohort, 17 of 31 patients with monogenic inflammatory bowel disease were candidates for allogeneic stem cell transplant as the only curative option ^35^. Ashton *et al.* found NOD2variants in 20 patients, *TRIM22* variants in five patients, and a variant related to the Wiskott-Aldrich syndrome in four patients. Additional monogenic variants were in XIAP, MASP2, and NCF2, among others ^38^. Lesga *et al*. also found mutations related to the Wiskott-Aldrich syndrome), and *CD40L, FOXP3, CYBA, CYBB, TTC37*, and *DKC1* in twelve patients with very early onset inflammatory bowel disease ^39^.

Additionally, Ashton *et al*. described a cohort of 411 patients with inflammatory bowel disease, with a mean age of 11.92 years (range: 1.3 -17.39). In their cohort, they found 11.5% variants in a known monogenic inflammatory bowel disease, with the highest percentage in the very early- onset presentation group (16.7%). They described that Crohn’s disease, and its stricture phenotype (68.4%) significantly increased the risk of undergoing intestinal resection (63.2%), more severe and variable disease phenotype, and severe ulcerative colitis with liver disease, depending on the associated genetic variations ^38^. Similar findings were also found in the multicenter observational study conducted by Lega *et al*., in Rome, Italy, in selected patients with very early-onset and early-onset inflammatory bowel disease with severe or atypical phenotypes. They found 12 patients with gene variants associated with more severe or atypical manifestations compared to the group without inborn errors of immunity ^39^.

For the five case reports and one case series, the following findings were documented: Ziv *et al*. described a 12-month-old female patient with severe ulcerative colitis within the first year of life. They highlighted a history of prematurity, failure to thrive, dysmorphic features, and severe Pneumocystis proved pneumonia. A genetic study identified a mutation in RTEL associated with dyskeratosis congenita ^40^. Likewise, Malik *et al*. reported the case of a 16-year-old girl with inflammatory bowel disease associated with a history of complex autoimmune and inflammatory disorders, recurrent episodes of prolonged bronchitis, chronic recurrent middle ear infections, and a family history of hematologic malignancies and autoimmune manifestations. A whole exorne sequencing study revealed a heterozygous variant in *TNFRSF13B*^41^.

In the articles about monogenic inflammatory bowel diseases in adults, Vardi *et al*. described the case of a 37-year-old male with atypical symptoms of inflammatory bowel disease since he was 15 years old, highlighting the presence of two siblings with gastric adenocarcinoma developed at a young age, consanguineous parents, multi-organ involvement associated with polyarthritis, vitiligo, sensory-motor neuropathy, and hepatitis of unknown origin. Single whole exorne sequencing found an *LRBA* mutation ^42^. Edwards *et al*. showed the case of a 26-year-old man with early- onset immune dysregulation and autoimmune hemolytic anemia, which required splenectomy due to refractoriness to first and second line of immunomodulatory treatment, with inflammatory bowel disease resistant to conventional therapies and antibody deficiency. They found a novel homozygous *LCP2* variant ^43^.

Autoinflammatory disorders may be misdiagnosed as autoimmune diseases. Olyha *et al*. described 14 male patients with an early diagnosis of inflammatory bowel disease with clinical characteristics of Behçefs disease ^44^. These patients had a deficiency of *ELF4*, X-linked (DEX), caused by a hemizygous loss-of-function mutation in the E74-like ETS transcription factor 4 (*ELF4*) in the X chromosome. Evaluated patients had a mean age of 4.5 years at disease onset. Most of them presented with symptomatic flares lasting 2-5 days, consisting of oral ulcers on the tongue, uvula, lips, and hard palate. Other commonly reported symptoms were fever, arthritis, abdominal pain, and diarrhea ^44^. Most patients had poor weight gain or loss. Of 14 patients, 7 underwent upper and lower endoscopy with histology findings of aphthous-like ulcers; the histologic findings showed inflammatory infiltrate with lymphocytes and neutrophils but without granuloma formation ^44^. Most patients had a previous diagnosis of Crohn’s disease or unclassified inflammatory bowel disease as they did not fulfill the diagnostic criteria for proper inflammatory bowel disease. Most patients experienced tender papulonodular lesions during symptomatic flares and pathergy. The biopsy revealed lobular and septal panniculitis. The final diagnosis was made by whole exorne sequencing and Sanger sequencing ^44^ (table 2).

## Discussion

This review emphasizes the importance of high suspicion of inborn errors of immunity in pediatric and adult patients with autoimmune diseases. Autoimmune cytopenias are one of the diseases with the strongest association with inborn errors of immunity; the relationship between both is well documented. In some cases, almost half of the patients with autoimmune cytopenias such as autoimmune hemolytic anemia, autoimmune thrombocytopenia, or Evans’ syndrome, have variants in genes like FAS and *CTLA4*^9^. In 2017, Azizi *et al*. found autoimmune disorders as the first presentation of immunodeficiency in 2.5% of adults with primary antibody deficiencies. Autoimmune thrombocytopenia and autoimmune hemolytic anemia were the most frequent first clinical presentations, mainly in common variable immunodeficiency ^15^. Autoimmune cytopenias may be secondary to infection or autoimmune disease. However, when they become chronic, recurrent, or refractory to first-line treatments or associated with a personal or family history of autoimmunity, an early disease onset of inborn errors of immunity must be ruled out ^12^^,^^28^^,^^45^. Inborn errors of immunity, autoimmune manifestations, chronic lymphoproliferation, or unusual infections may develop over the years, complicating an early diagnosis due to an incomplete phenotype in the initial evaluation ^15^. In adults, granulomatous inflammation in the absence of sarcoidosis requires a complete evaluation of the immune system, since this is a frequent finding in common variable immunodeficiency ^46^.

Besides common variable immunodeficiency, autoimmune lymphoproliferative syndrome is one of the most important etiologies of autoimmune cytopenias. Autoimmune lymphoproliferative syndrome is characterized by defective lymphocyte homeostasis, usually due to failed Fas-mediated apoptosis. Acquired or inherited genetic variants leading to defects in the classic Fas-Fas ligand pathway result in abnormal lymphocyte survival, causing benign and chronic (more than 6 months) non-infectious lymphoproliferation, autoimmunity, and increased lymphoma risk ^47^. Specific tests for autoimmune lymphoproliferative syndrome should include vitamin B12 levels (abnormal > 1,500 ng/L) and the presence of doublenegative T lymphocytes ^20^^,^^48^.

Systemic lupus erythematosus is a chronic, multisystem inflammatory disease of autoimmune etiology. When it occurs before age 18, it is called childhood-onset systemic lupus erythematosus ^29^. Although clinical and laboratory characteristics are usually similar in adult and pediatric patients, children typically have greater organ involvement and a more severe presentation ^30^. Furthermore, pediatric patients teach us that clinical data can guide us when faced with a monogenic disorder that explains the appearance of the disease. Reports from China and India suggest that systemic lupus erythematosus should be suspected in those patients with clinical manifestations before the age of 5, with atypical presentations such as extensive mucocutaneous, joint, renal, and neuropsychiatric involvement, and who also have consumption of complement proteins without other signs that suggest disease activity. In these patients, monogenic variants in genes related to systemic lupus erythematosus usually cause the disease ^29^^,^^30^.

Other clinical data that may suggest monogenic disease as the cause of systemic lupus erythematosus include hematological alterations, such as immune-mediated cytopenias, mainly thrombocytopenia and Coombs-positive hemolytic anemia. These alterations do not improve with conventional therapy and are associated with recurrent infections or chronic lymphoproliferation, as reported in the case series by Wang *et al*. and the cohort of Li *et al*. ^31^^,^^32^. Monogenic disease should also be suspected in patients with skin lesions suggestive of lupus chilblain at the time of presentation, as reported in the case series by Yarbrough *et al*. ^33^, and when patients present with atypical signs of the disease that point to immune deregulation ^34^.

Inflammatory bowel disease is diagnosed in adult and pediatric populations. Its onset in adults has a low suspicion rate of monogenic origin. In children, most information is about patients with very early-onset inflammatory bowel disease due to their association with more severe clinical courses, more extensive intestinal inflammation, higher rates of complications, and treatment resistance ^37^. Pediatric monogenic inflammatory bowel disease patients teach us the main warning signs to conduct genetic and immunologic studies promptly. Monogenic inflammatory bowel disease typically presents early in life in the cohorts evaluated. Most genetic inflammatory bowel disease patients have symptom onset before two years of age, associated with extraintestinal manifestations (such as arthritis or folliculitis in patients with *IL 10R* deficiency). Additionally, we found a higher rate of parental consanguinity and a family history of autoimmune disease ^37^. Monogenic inflammatory bowel disease patients have higher rates of severe perianal/fistulating disease, linear serpentine ulcerations, and non-caseating granulomas. They are likely to be diagnosed with Crohn’s disease or unclassified inflammatory bowel disease ^37^. One of the most important red flags is the need for surgical intervention in pediatric inflammatory bowel disease patients ^36^. In adult-onset, information is scarce about the monogenic inflammatory form of the disease, case report number, and performed studies. However, certain clinical and paraclinical clues raise clinical suspicion in this population, such as a personal history of recurrent infections, resistance to conventional therapies, atypical symptoms of inflammatory bowel disease, multiorgan involvement associated with the manifestations of poly-autoimmunity, abnormal findings in immunological workup, atypical endoscopic or histological findings, young age at presentation, family history of neoplasms and autoimmune manifestations, and consanguineous parents ^41^^,^^42^ (table 2).

In summary, the immune system study should include the quantitative and qualitative evaluation of T and B cell subpopulations or the innate system when a specific diagnosis is suspected in autoimmune diseases. It is crucial to exclude secondary causes such as protein loss, hematologic malignancies, and certain medications, to diagnose primary antibody immunodeficiencies or humoral defects in patients with hypogammaglobulinemia ^27^. The functional study of the humoral response should include antibody assessment, including IgG to T-dependent vaccines such as tetanus, diphtheria, rubella, and mumps. T-independent response requires measuring IgG to the 23-valent pneumococcal vaccine ^49^. The initial cellular quantitative evaluation includes measuring T lymphocytes (CD3+, CD4+, CD8+), B lymphocytes (CD19+, CD20+), and natural killer cells (CD16+, CD56+), and the functional assessment should include lymphoproliferation studies ^50^.

The number of potential candidate genes and overlapping phenotypes highlights the importance of genetic tests in patients with autoimmune and suspected monogenic diseases, regardless of age ^10^. Genetic testing returns high rates of variants of unknown significance, highlighting the need for expert interpretation and standardized variant analysis. Some primary immunodeficiency disorders in adults, such as common variable immunodeficiency, are predominantly polygenic, although recent studies suggest an increase in monogenic forms ^19^^,^^22^^,^^27^. Incomplete penetrance and variable expressivity of variants suggest that additional genetic, epigenetic, and environmental factors influence disease development ^27^. However, making a genetic diagnosis helps the clinician foresee potential complications, provide a prognosis, and target therapy ^15^. One of the most critical aspects of genetic diagnosis is counseling patients and their families, as most will adjust their reproductive plans according to the information received.
